# Integrating Network Pharmacology, Molecular Docking and Experimental Validation to Explore the Pharmacological Mechanisms of Quercetin Against Diabetic Wound

**DOI:** 10.7150/ijms.100468

**Published:** 2024-10-28

**Authors:** Zhe Zhang, Lei Wang, Xuan Li, Yuxi Miao, Dongyu Li

**Affiliations:** 1 Department of General Surgery & VIP In-Patient Ward, The First Hospital of China Medical University, Shenyang, Liaoning Province, 110001, China.; 2 Department of Vascular and Thyroid Surgery, The First Hospital of China Medical University, Shenyang, Liaoning Province, 110001, China.; 3 Department of Pharmacology, School of Pharmacy, China Medical University, Shenyang, Liaoning Province, 110122, China.

**Keywords:** quercetin, diabetic wound, polyphenol, PI3K-AKT signaling, molecular docking, network pharmacology

## Abstract

The chronic non-healing diabetic wound (DW) has remained a challenge to both the society and individuals. Previous studies suggested dietary moderate consumption of quercetin (QCT) are beneficial in preventing diabetic complications, including non-healing DW. However, there were few studies that have investigated QCT-related underlying molecular mechanisms against DW. In the present study, we for the first-time combined network pharmacology with molecular docking and experimental validation to investigate QCT-related therapeutic targets and mechanisms for treating DW. Finally, 191 QCT-related targets and 1750 DW-related pathogenetic targets were obtained from online databases. After removing duplicates, a total of 90 potential therapeutic targets of quercetin for treating DW were ultimately identified. Furthermore, 7 targets with higher degree including IL-6, EGFR, SRC, TNF, AKT1, JUN and MMP9 were predicted as central therapeutic targets of QCT for treating DW. Functional enrichment analysis demonstrated that QCT exerted strong levels of multitargeting regulatory activity. In addition, the KEGG enrichment analysis indicated that several signaling pathways including AGE-RAGE signaling pathway in diabetic complications, IL-17, PI3k-AKT, TNF, HIF-1, VEGF were predicted as key regulators of QCT for treating DW. Molecular docking results suggested that QCT had strong binding activity with the predicted targets. In addition, verification experiments suggested that QCT could significantly attenuated the expression of inflammatory cytokines and the regulation of PI3K-AKT signaling pathway was probably a vital mechanism involved in the pharmacological mechanism of QCT for treating DW. Taken together, combined network pharmacological with experimental validation, we for the first time systematically investigated associated-therapeutic targets and potential pathways of QCT for DW treatment. Our study might provide theoretical basis for DW treatment.

## Introduction

Diabetes mellitus (DM) is a serious, costly metabolic disorder with high morbidity. Despite improving living standards, the prevalence of DM has seen an explosive increase in the past few decades 1. The heavy cost of diabetes and related complications have become a significant burden to society 2. Clinically, DM patients are generally asymptomatic in the early stages. Unfortunately, without available medications, it may lead to serious damage and even cause death 3. Diabetic foot ulcer (DFU) and refractory wounds are serious chronic complications of DM, contributing to over 84% of DM-related lower limb amputations 4. The normal wound healing process can be divided into 3 overlapping and dynamic phases: inflammation, proliferation, and tissue remodeling 5. However, the chronic refractory wound healing in diabetic patients is a complex and long-term process 6. Under pathological conditions, chronic hyperglycemia negatively affects the normal healing process, triggering the activation of inflammation cascades 7. Currently, medical therapies against refractory wounds are limited. Therefore, it is of great importance to explore novel therapies for diabetic refractory wounds.

Plant-derived natural products have gained much attention due to their affordability, safety and lower side effects 8. Quercetin (QCT), one of the most well-studied phytochemicals, belongs to the subclass of flavonoids and is widely abundant in fruits and vegetables, such as apples, onions, peppers and berries 9. Previous studies suggested that moderate consumption of QCT is beneficial to health. Accumulating evidence has shown that QCT has various health-prompting properties such as anti-inflammation, anti-oxidant, anti-tumor and cardioprotective capabilities 10. The role of QCT play in wound healing is well-studied. Numerous studies suggested that QCT is a promising natural wound healing agent 11. A recent study published by Harvinder suggested that QCT-loaded AgNPs in hydrogel matrices significantly attenuated wound gaps in an experimental DW model 12. Furthermore, Vinay reported that QCT ointment obviously reduced expression of pro-inflammatory cytokines and improved angiogenesis in DW rats 13. All these findings suggested that quercetin is a promising drug in accelerating DW healing.

Network pharmacology is considered the next paradigm of drug discovery and is helpful in screening potential therapeutic targets and pathways from a molecular perspective 14. In the present study, network pharmacology methods combined with molecular docking was utilized to explore potential therapeutic targets and underlying mechanisms of QCT for treating DW. Furthermore, experimental validations were performed to testify our predictions. Our study might provide theoretical basis for DW research.

## Materials & Methods

### Screening for QCT-related therapeutic targets against DW

In this study, QCT-related targets were obtained from the Traditional Chinese Medicine Systems Pharmacology database 15 (TCMSP, https://old.tcmsp-e.com/index.php), the Encyclopedia of Traditional Chinese Medicine database 16 (ETCM, http://www.tcmip.cn/ETCM/index.php), and the SwissTargetPrediciton database (http://www.swisstargetprediction.ch/index.php). Furthermore, DW-related targets were obtained from the Therapeutic Target database 17 (TTD, https://db.idrblab.net/ttd/), the Online Mendelian Inheritance in Man database 18 (OMIM, https://omim.org/), the GeneCards database (https://www.gene-cards.org/) and the Disgenet database 19 (https://www.disgenet.org/). Then the drug and disease-related targets were summarized and removed the duplicates. The Venn diagram (http://bioinformatics.psb.ugent.be/webtools/Venn/) was used to overlap all the non-duplicates targets which were seen as potential therapeutic targets in QCT-related DW treatment.

### Construction of protein-protein interaction (PPI) network and screening for potential therapeutic targets of QCT for DW treatment

To find protein-associated networks of QCT against DW, the overlapped genes were imported into the STRING Database (https://cn.string-db.org/). In this network, we set the minimum required interaction score as high confidence (0.700) and the species was set as Homo sapiens. Then, the Cytoscape software (Version 3.9.1) was used to visualize the PPI network. Then the Cytohubba plugin was used to explore core therapeutic targets of QCT against DW.

### Biological enrichment analysis and construction of gene-pathway network

In this study, the Gene Ontology (GO) in biological processes (BP), molecular function (MF) and cellular components (CC) and Kyoto Encyclopedia of Genes and Genomes (KEGG) pathways were performed by using the Metascape database(https://metascape.org/gp/index.html). Furthermore, we used the Bioinformatic database (http://www.bioinformatics.com.cn/) to visualize identified results. Then we used Cytoscape software to construct the gene-pathway network.

### Molecular docking

To better identify molecular interactions behind the ligand and macromolecules, molecular docking analysis was performed in this study. The PubChem database (https://pubchem.ncbi.nlm.nih.gov/) was used to retrieve the structure of QCT and identified core targets. Then we used Pymol software (https://py-mol.org/2/) to remove water and unwanted atoms. The Autodock (https://autodock.scripps.edu/) was used to perform molecular docking procedures. The Pymol software was used to visualize the binding site between the ligand and macromolecules. The DiscoveryStudio 2019 software was used to visualize molecular bonds between QCT and identified core targets.

### Experimental DM model establishment

The streptozotocin (STZ) was obtained from Sigma-Aldrich (United States). The citric acid/sodium citrate buffer were obtained from Beyotime Biotechnology (Shanghai, China). 30 8-week-old, 280-300g male Sprague Dawley (SD) rats were obtained from Liaoning Changsheng Biotechnology (Liaoning, China) and were housed under controlled environment with sufficient food and water. The whole experimental animal protocols were approved by the Institutional Animal Care and Use Committee of China Medical University (Ethic number: CMU20241591). And the animal experimental procedures were performed following the guideline of China Medical University Guide for the Care and Use of Laboratory Animals. After one-week of adaptive feeding, 30 SD rats were randomly divided into 3 groups (n=10): control group, STZ-induced DW group and QCT-treated group. Rats in STZ-induced DW group and QCT-treated group were injected intraperitoneally with 65mg/kg STZ (dissolved in 0.1mol/L citric acid/sodium citrate buffer, PH=4.2) to induce diabetic model 20. Rats in control group were injected intraperitoneally with equal amounts of 0.1mol/L citric acid/sodium citrate buffer without STZ. To prevent hypoglycaemia, 5% glucose was added to the drinking water at the day of diabetes induction. After diabetes induction, the rats' blood glucose levels were measured for consecutive 4 weeks. The rats with blood glucose level greater than 16.7mM were deemed as diabetes rats and were used for the experiment.

### Experimental DW model establishment and QCT administration

QCT was purchased from Shanghai Yuanye Bio-Technology Co., Ltd (B20527, Shanghai, China). QCT was suspended in 0.5% sodium carboxymethyl cellulose. The fresh QCT solutions were prepared daily. To establish DW model, after 4 weeks of STZ injection, diabetic rats were anesthetized with 2% isoflurane and the dorsal hair were shaved. After the surgical site was disinfect with 75% alcohol, a 3*3cm full-thickness excisional wound was created on their backs. To avoid skin contraction, the wound site was stabilized with 4-0 nylon sutures. After surgery, rats in QCT-treated group were administered with 40mg/kg QCT solution by oral gavage daily and rats in other group were treated with equal amounts of solution without QCT. The dose of QCT was according to previously published work 21. The drug treatments continued for 3 weeks. After 3-week treatment, all rats were euthanized by excessive anesthesia (5% isoflurane) and skin tissues were collected for further analysis. To record the healing process, the photos of wound area were taken at days 0, 5, 10, 15, 20 after surgery. The wound area was measured and the wound healing rate was analyzed by the imageJ software. The wound healing rate was represented as (original wound area - daily wound area) / (original wound area) * 100%.

### Western blotting

In the present study, the skin tissues were collected. The total protein extraction kit was obtained from Solarbio, China to extract proteins from skin tissues. The blotting procedures were performed to identify characterization of predicted targets as previously described 22. Furthermore, the imageJ and Graphpad Prism software was used to analyzed and visualized the image. The primary antibodies used in this study were as follows, p- indicated phosphorylation. Antibody against MMP9(No: ab76003), TNFα (No: ab34674), IL6 (No: ab259341) and β-actin (No: ab8227) was obtained from Abcam (Cambridge, MA). Antibody against mTOR (No.2983), p-mTOR (No.5536), AKT(No.4691), p-AKT (No.9271), PI3K (No.4249), p-PI3K (No.4228) was obtained from cell signaling technology (CST).

### Histological study of wound sections

The wound tissues were collected and fixed in 4% paraformaldehyde and then embedded in paraffin. The tissues were sectioned into 5μm thickness slides, and stained by HE (haematoxylin and eosin), Masson and Immunohistochemical (IHC) protocols. The stained procedures were performed as previously described 22. The level of TNF, IL6, p-PI3K, p-AKT and p-mTOR were assessed with IHC staining. The ImageJ software was used to qualify the stained area.

### Statistical analysis

Quantitative data from 3 independent experiments were expressed as mean ± SEM. These data were analyzed by using Statistical Product Service Solutions (SPSS, Version 26) and visualized by GraphPad Prism software (Version 9.0.0). In addition, the two-way Analysis of Variance (ANOVA) was performed to compare the differences. P < 0.05 was considered to be statistically significant.

## Results

### Screening for potential therapeutic targets of QCT for DW

**Figure 1:**
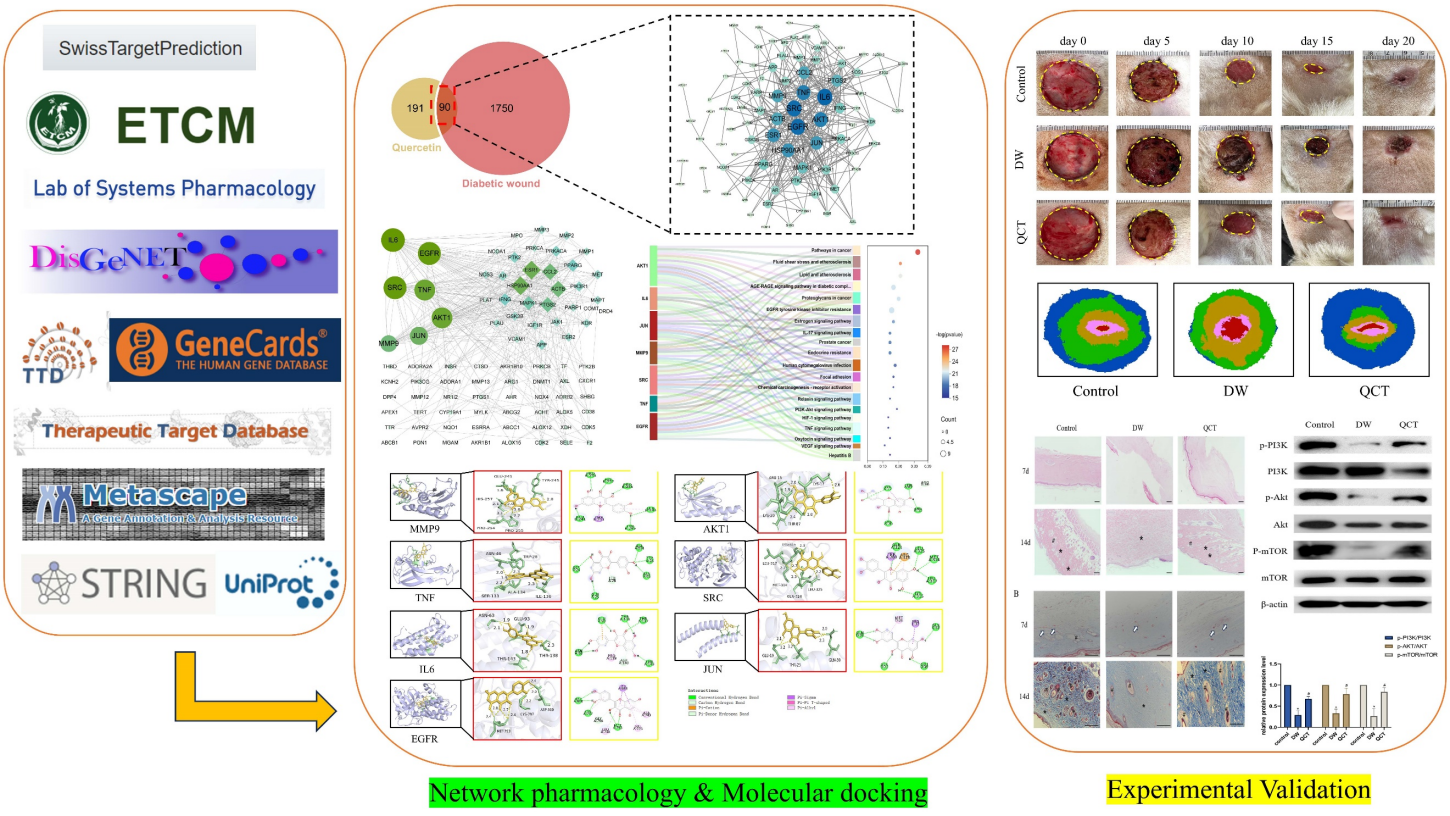
The workflow of the present study.

**Figure 2:**
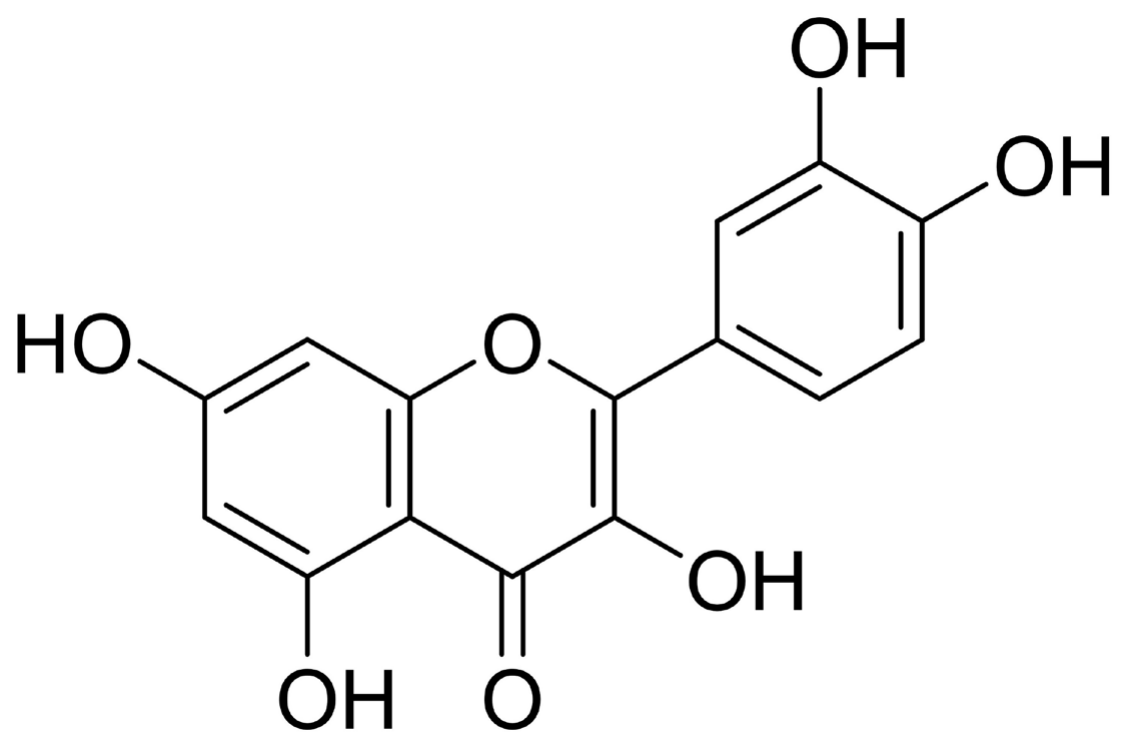
The chemical structure of QCT.

**Figure 3:**
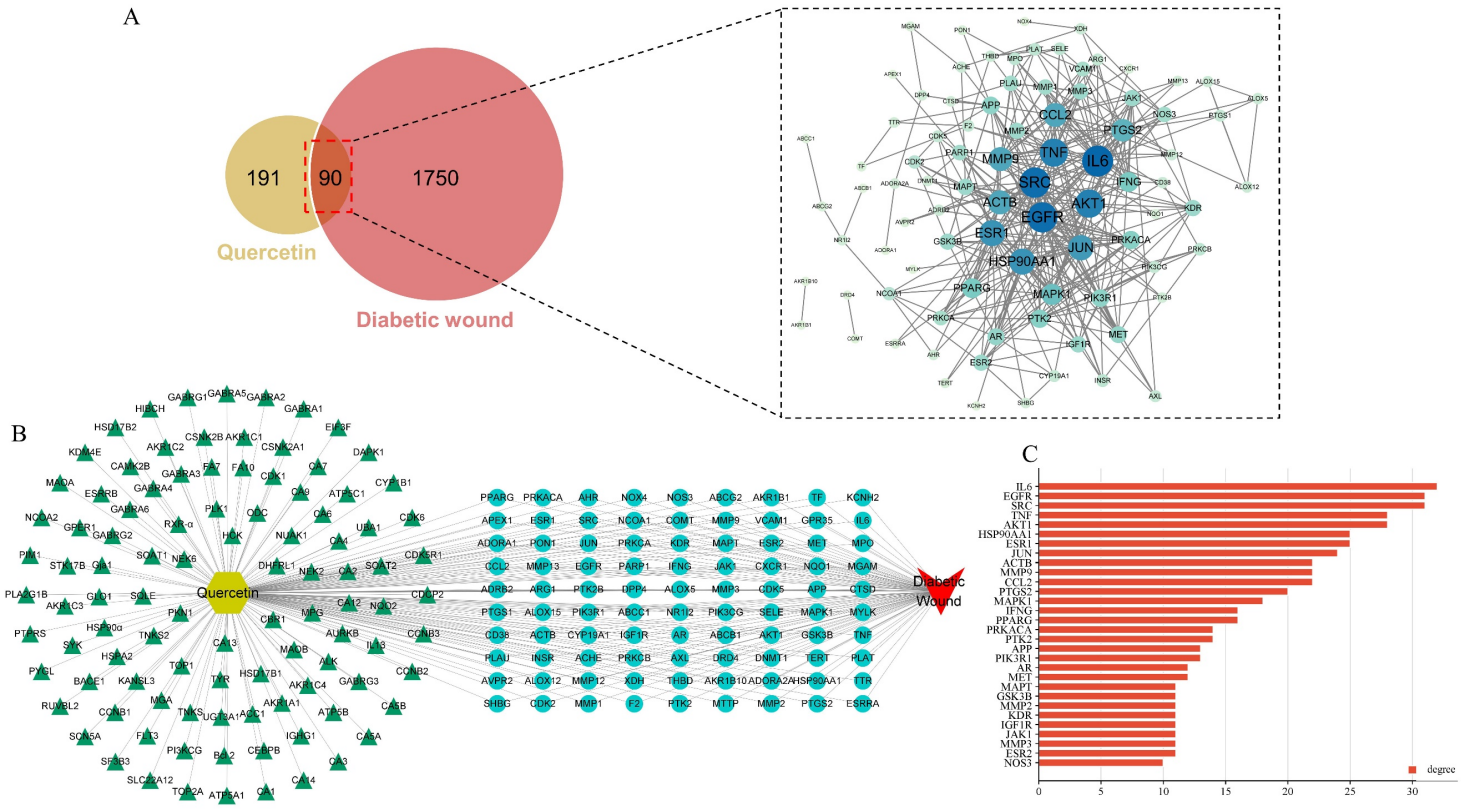
Network pharmacology predicted therapeutic targets of QCT for treating DW and PPI network construction. (A)Venn diagram of QCT and DM-related targets. The intersection of the yellow circle and red circle represented the intersecting targets. (B) Predicted therapeutic targets of QCT for treating DW. (C)The bar chart representing the top 30 targets ranked by degree.

The work flow of the whole study is presented in Figure 1. The chemical structure of QCT was depicted in Figure 2. As shown in Figure 3a, a total of 191 QCT-related targets were obtained from the intersection of TCMSP, ETCM and SwissTarget database after merging and deleting duplicates. Besides, 1750 DW-related targets were obtained from the TTD, the Disgenet, the Genecards, and the OMIM database. Then, identified targets of QCT and DW were overlapped with a Venn diagram and constructed a drug-disease network. As shown in Figure 3b, a total of 90 potential therapeutic targets of QCT for treating DW were ultimately identified. The overlapped targets were shown in Table 1 and were considered potential therapeutic targets for QCT against DW.

### Identification of core targets of QCT against DW

**Figure 4:**
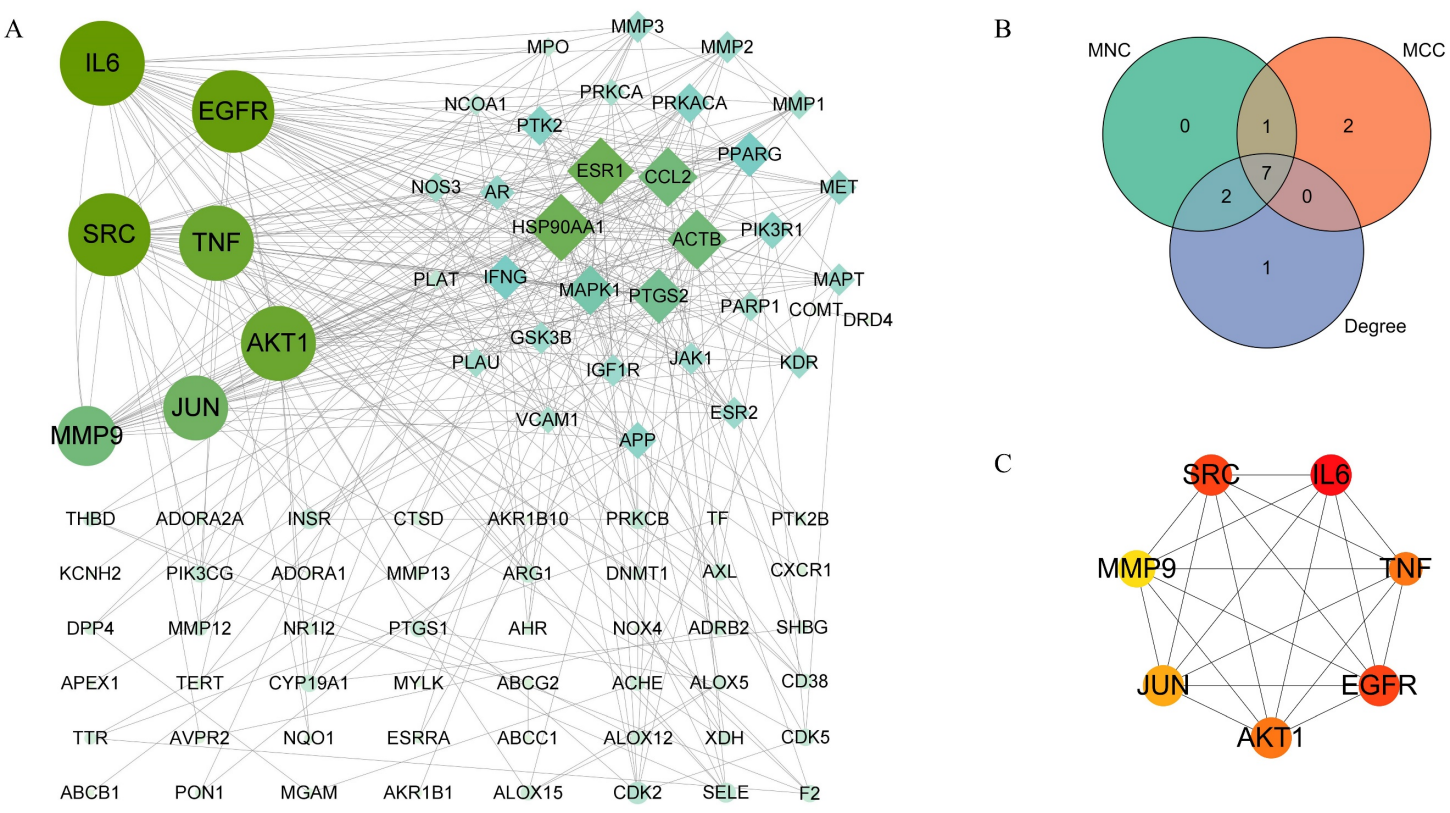
Identification of core therapeutic targets of QCT against DW. (A) Citespace analysis representing the overlapping targets. (B) Venn diagram of intersection among MNC, MCC and degree. (C) Top 7 core targets. Larger and darker represented higher degree.

To explore core targets involved in QCT-related DW treatment, the PPI network was constructed and was visualized by using the Cytoscape software. As shown in Figure 4A, the larger nodes with deeper color indicated they had more influential. To ensure the accuracy, three prediction methods, the maximal clique centrality (MCC), maximum neighborhood component (MNC), and degree method, was used in this study. Then, we overlapped the prediction results and 7 targets in common were obtained (Figure 4B). As shown in Figure 4C, these targets were interleukin 6 (IL-6), epidermal growth factor receptor (EGFR), proto-oncogene tyrosine-protein kinase Src (SRC), tumor necrosis factor (TNF), RAC-alpha serine/threonine-protein kinase (AKT1), transcription factor jun (JUN) and matrix metalloproteinase-9 (MMP9). The degree of predicted core targets was listed in Table 2 and were chosen for further analysis.

### Functional enrichment analysis

**Figure 5:**
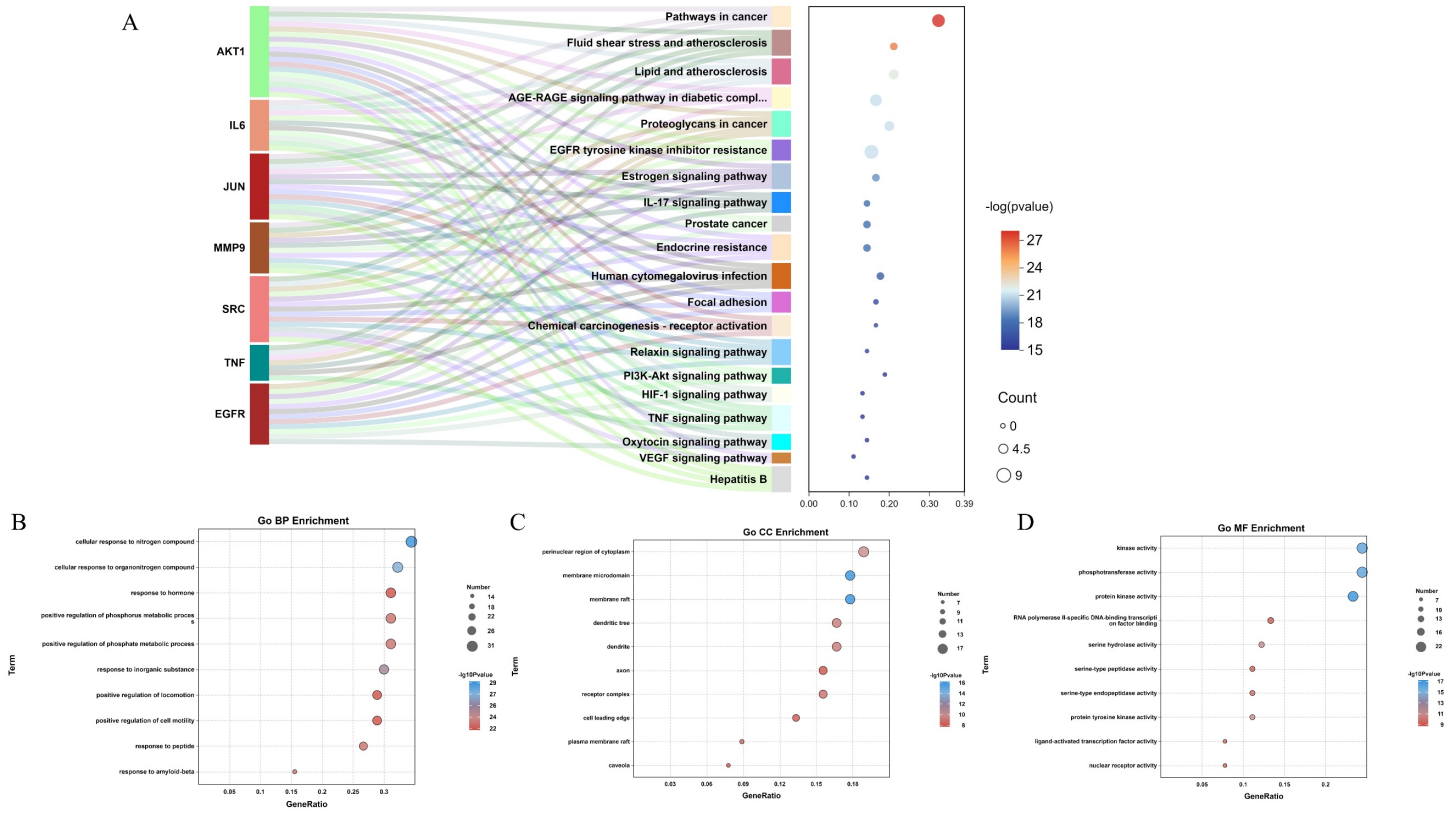
Functional enrichment analysis. (A) The KEGG enrichment analysis. (B-D) The GO enrichment analysis (BP, CC and MF). The node size represented the number of targets annotated to the term. The color of nodes represented the p value.

To better investigate the functional mechanism and pathways of QCT for treating DW, the predicted targets were uploaded to the Metascape database for enrichment analysis. In the present study, a total of 1419 GO terms and 178 KEGG pathways were obtained. The Sankey diagram summarized the contribution between identified core targets and enriched KEGG pathways (Figure 5A). The enriched GO biological process (BP), cellular components (CC) and molecular function (MF) was shown in Figure 5B, 5C and 5D respectively. The top 10 GO processes were listed in Table 3 and the top 20 KEGG pathways were shown in Table 4. In view of the results, we found that QCT affected a serious of biological processes including cellular response to nitrogen compound, cellular response to organonitrogen compound, response to inorganic substance, positive regulation of phosphate metabolic process, positive regulation of phosphorus metabolic process *et al.* Moreover, we found that the most significantly enriched KEGG pathways were pathways in cancer, fluid shear stress and atherosclerosis, Lipid and atherosclerosis, AGE-RAGE signaling pathway in diabetic complications, proteoglycans in cancer. And the most relevant pathway involved in QCT for treating DW might be the AGE-RAGE signaling pathway in diabetic complications.

### Molecular docking analysis

**Figure 6:**
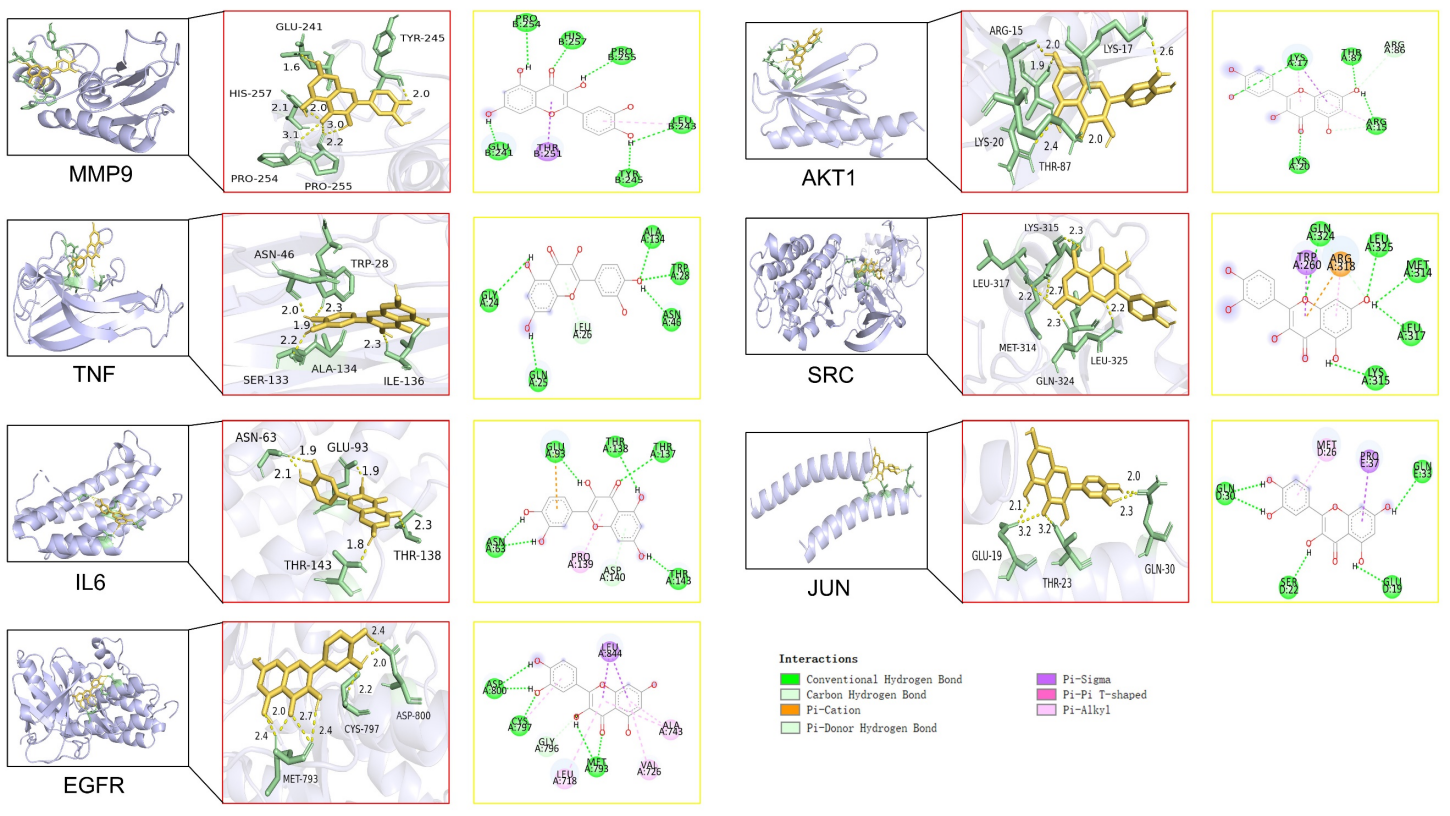
Molecular docking of QCT with identified core targets.

In this study, molecular docking analysis were performed to evaluate the binding sites and binding energies between the QCT and macromolecules (Figure 6). The crystal structures of ligands, possible binding sites and binding energies were shown in Table 5. We found that the binding energies of QCT with core macromolecules ranged from -3.75 to -7.35. These results indicated that QCT had better affinities with these macromolecules. In view of our results, we found that QCT had the strongest affinity with MMP9 by possessing six conventional hydrogen bonds with PRO254, HIS257, PRO255, LEU243, TYR245 and GLU241; one Pi-Sigma interaction with THR251 and one Pi-Alkyl interaction with LEU243. As for TNF, QCT possessed 5 conventional hydrogen bonds with GLY24, ALA134, TRP28, ASN46, GLN25 and one Pi-Donor Hydrogen Bond with LEU26. Besides, QCT had strong affinity with IL6 by forming 6 conventional hydrogen bonds with GLU93, THR138, THR137, THR143 and ASN63; one Pi-cation interaction with GLU93; one Pi-Donor Hydrogen Bond with ASP140 and one Pi-Alkyl interaction with PRO139. As for EFGR, QCT formed 5 conventional hydrogen bonds with MET793, CYS797, ASP800; three Pi-Alkyl interactions with ALA743, VAL726 and LEU718; and one Pi-Sigma interaction with LEU844. As for AKT1, QCT formed 4 conventional hydrogen bonds with LYS17, THR87, ARG15 and LYS20; one carbon hydrogen bond with ARG15; two Pi-Alkyl interactions with LYS17 and ARG15; one Pi-Sigma interaction with LYS17. And QCT had affinity with SRC by forming 5 conventional hydrogen bonds with GLN324, Leu325, MET314, LEU317 and LYS315; one Pi-cation interaction, one Pi-Alkyl interaction and one carbon hydrogen bond with ARG318; one Pi-Sigma interaction with TRP260. As for JUN, QCT formed 4 conventional hydrogen bonds with GLN30, SER22, GLU19, GLN33; one Pi-Alkyl interaction with MET26; one Pi-Sigma interaction with PRO37. Taken together, our results showed that QCT had better affinity with predicted macromolecules and might exert therapeutic effects by forming these interactions against DW.

### Treatment with QCT accelerated DW healing *in vivo* by reducing the activation of proinflammatory cytokines and regulating the PI3K-AKT-mTOR signaling pathway

**Figure 7:**
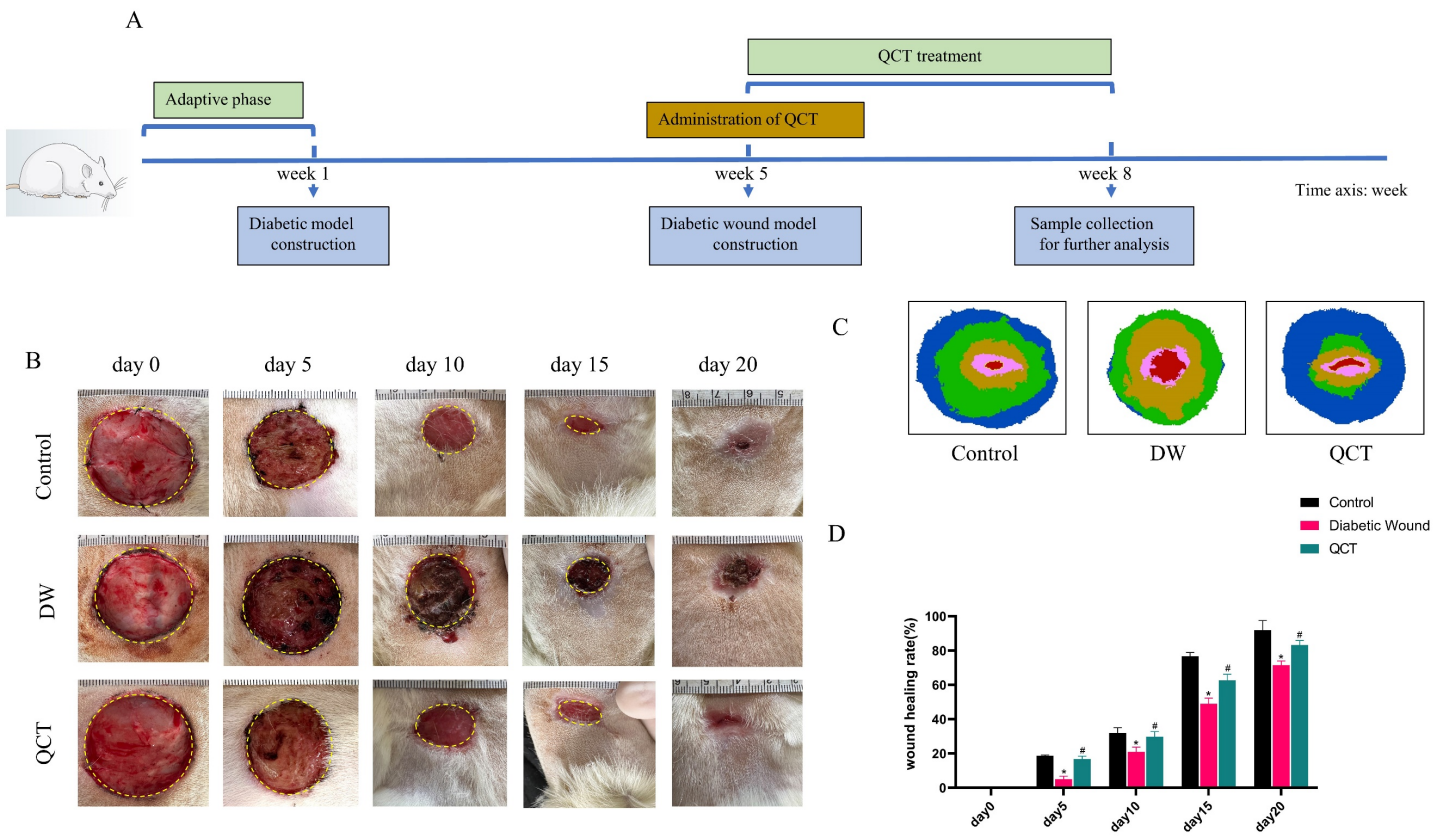
QCT accelerated DW healing in diabetic rats. (A) The flow diagram of animals. (B) The images of wound healing at different time points. (C) The overlay image of wounds in different group. (D) Statistical graph of wound healing rate. The wound healing rate=(S0-Sa)/S0*100%. S0 represented the original wound area (day 0) whereas Sa represented the wound area in day a. **p<0.01 versus control group, *p<0.05 versus control group; ##*p*<0.01 versus DW group, #*p*<0.05 versus DW group.

**Figure 8:**
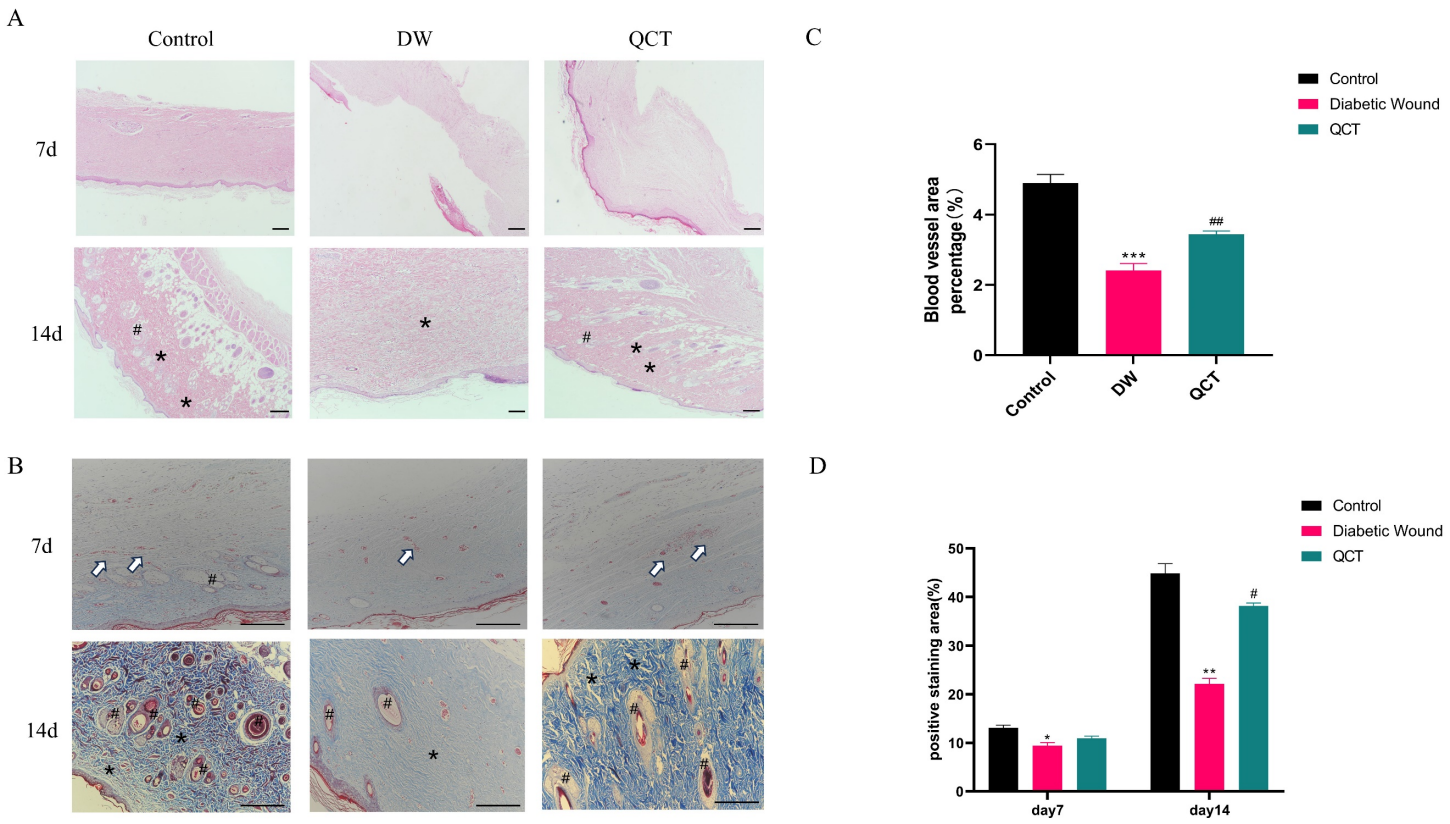
Photomicrographs of HE and Masson staining of wounds from different groups. (A) HE staining of wounds from different groups on days 7 and 10. (B) Masson staining of wounds from different groups on days 7 and 10. (C) The corresponding quantitative analysis of blood vessel percentage at early stage in different groups. (D) The corresponding quantitative analysis of collagen fiber in different groups. Data are represented as mean ± SD(n=3). Scale bar=100μm. The white arrow indicates blood vessel; * indicates collagen; # indicates skin appendage. **p<0.01 versus control group, *p<0.05 versus control group; ##p<0.01 versus DW group, #p<0.05 versus DW group.

The workflow of animals was shown in Figure 7A. The back wounds were photographed on the 0th, 5th, 10th, 15th, 20th after surgery (Figure 7B). Our results indicated that the diabetic wounds without treatment had the slowest healing rate compared to those in control and QCT group. Treatment with QCT promoted wound healing at different time points (Figure 7C). As shown in Figure 7D, compared with DW group, treatment with QCT significantly improved the wound healing rate. Then, we investigated the condition of wounds at early stages. As indicated in Figure 8A, by day 7, the wounds in control group were healing well. Skin samples in control and QCT group exhibited intact epidermis. Wound tissues from DW group had poorly regenerated epidermis. As shown in Figure 8B, treatment with QCT significantly increased the density of blood vessels. By day 14, the epithelialization process has appeared in all group. Administration of QCT increased the density of skin accessory organs such as blood vessels and hair follicles. In addition, treatment with QCT significantly increased collagen fiber deposition and the presence of blood vessels and hair follicles. The quantitative analysis was shown in Figure 8C and 8D.

**Figure 9:**
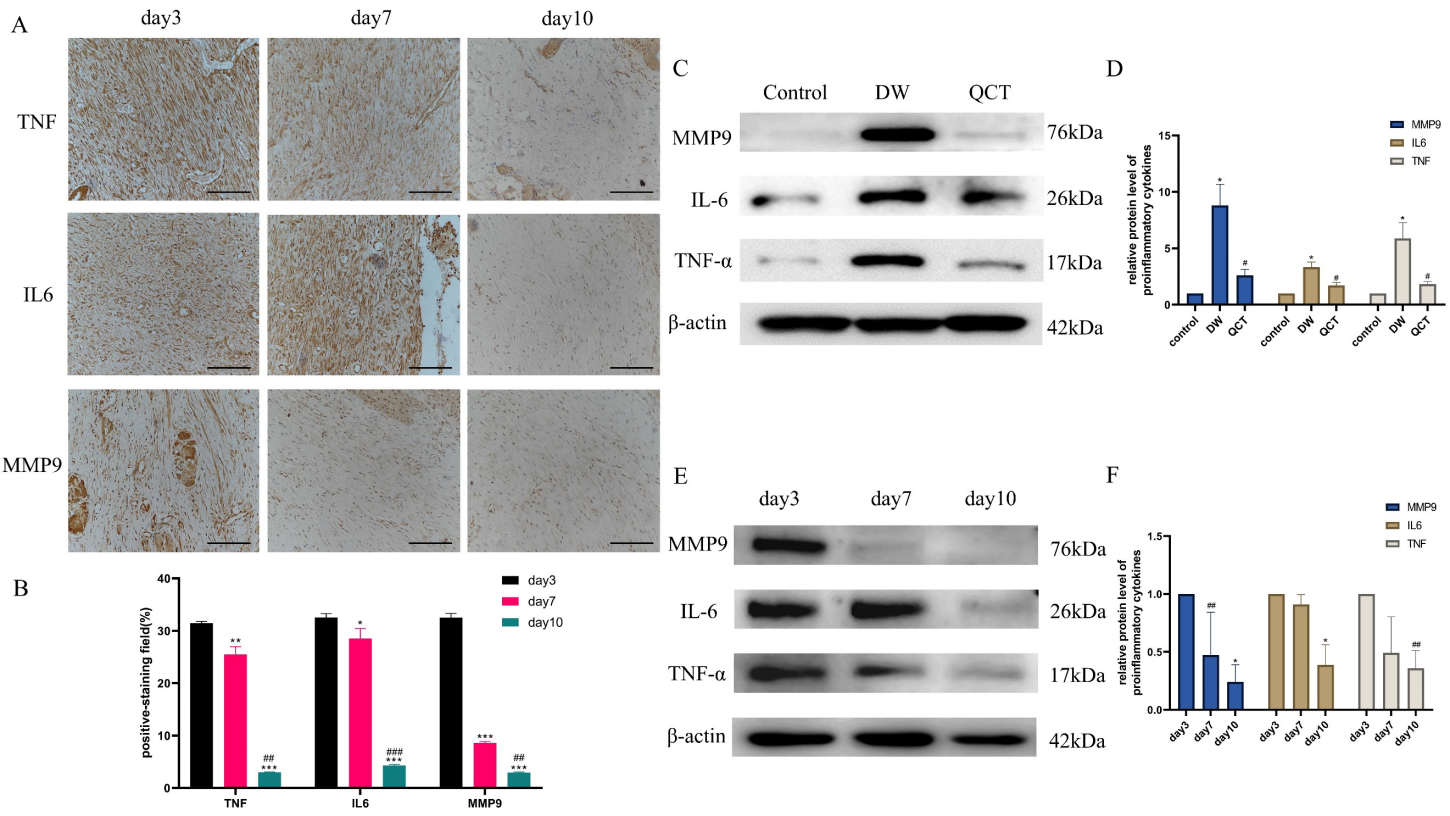
Effects of QCT on pro-inflammatory cytokines at early stages. (A) Immunohistochemical staining of TNF-α, IL6 and MMP9 at days 3, 7 and 10. Scale bar=50μm. (B) Quantitative analysis of TNF-α, IL6 and MMP9. (C) Representative WB images of MMP9, IL6 and TNF-α in different group. (D) Quantitative analysis of MMP9, IL6 and TNF-α in different group. (E) Representative WB images of MMP9, IL6 and TNF-α at days 3, 7 and 10. (F) Quantitative analysis of MMP9, IL6 and TNF-α at days 3, 7 and 10. ***p<0.001 versus day 3, **p<0.01 versus day 3, *p<0.05 versus day 3; ###*p*<0.001 versus day 7, ##*p*<0.01 versus day 7, #*p*<0.05 versus day 7.

**Figure 10:**
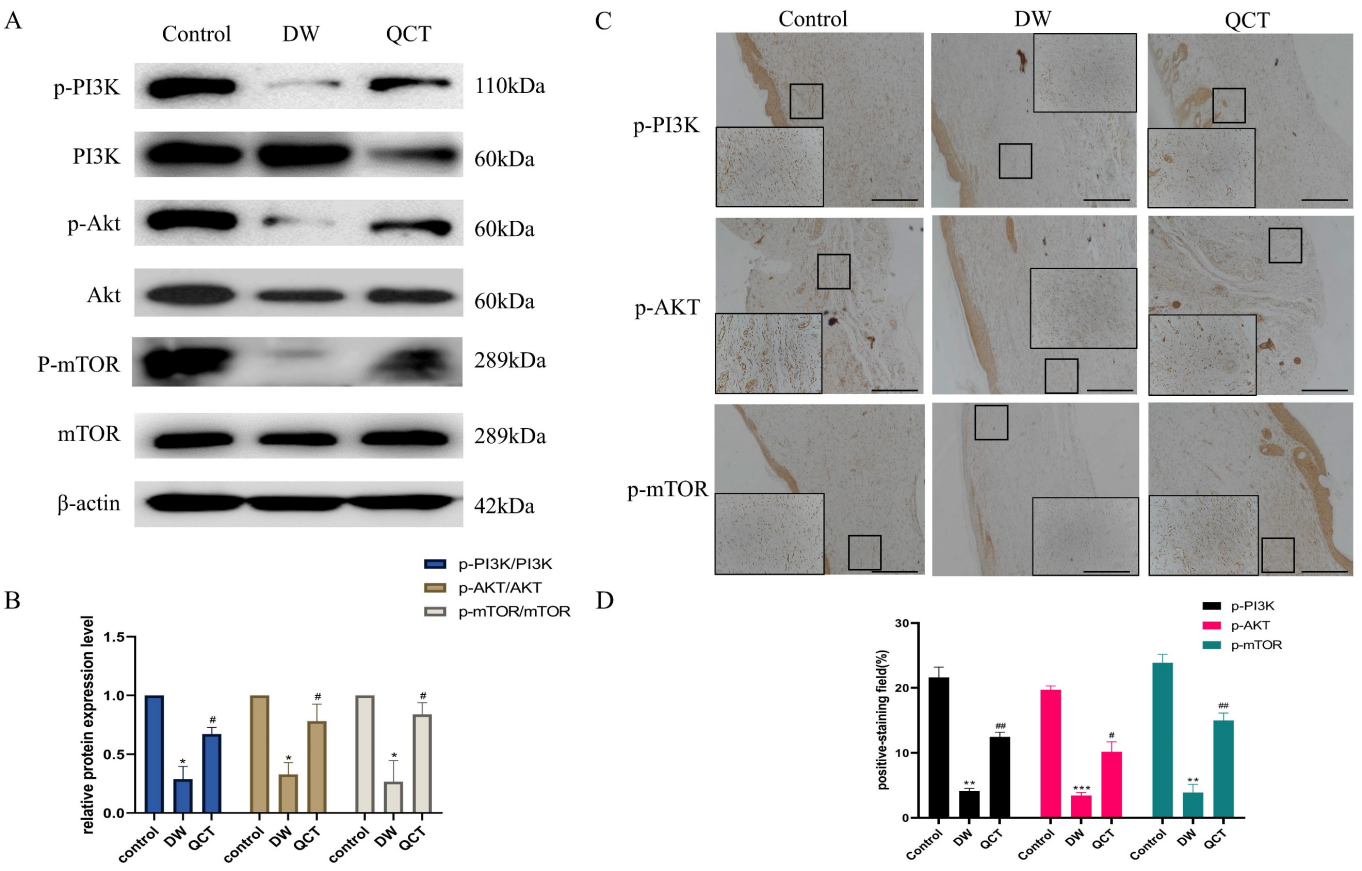
Effects of QCT on PI3k-AKT-mTOR signaling pathway. (A) Changes of PI3k-AKT-mTOR signaling pathway after QCT treatment were detected by WB. (B) Quantitative analysis of p-PI3K/PI3K, p-AKT/AKT, p-mTOR/mTOR. (C) Changes of PI3k-AKT-mTOR signaling pathway after QCT treatment were detected by immunohistochemical staining. Scale bar=50μm. (D) Quantitative IHC analysis of p-PI3K, PI3K, p-AKT, AKT, p-mTOR, mTOR. ***p<0.001 versus control group, **p<0.01 versus control group, *p<0.05 versus control group; ##*p*<0.01 versus DW group, #* p*<0.05 versus DW group.

To further investigate the role of QCT for treating DW, we collected the tissues from wound margin and performed WB and IHC analysis. The IHC and WB results demonstrated that treatment with QCT significantly attenuated the production of inflammatory cytokines IL-6, TNF-α and MMP9 at days 3, 7 and 10 (Figure 9A and 9E). The quantitative analysis was shown in Figure 9B and 9F. WB analysis showed that the protein expression of proinflammatory cytokines was consistent with IHC results (Figure 9C). The quantitative analysis was shown in Figure 9D. In addition, we investigated the role of QCT in the PI3K-AKT-mTOR signaling pathway. As shown in Figure 10A, treatment with QCT significantly increased the phosphorylation of PI3k, AKT and mTOR. The IHC staining showed the expression of the above proteins was consistent with WB results (Figure 10C). The quantitative analysis was shown in Figure 10B and 10D respectively. Taken together, these results suggested that QCT might exert beneficial effects against DW by reducing the activation of proinflammatory cytokines and regulating the PI3K-AKT-mTOR signaling pathway.

## Discussion

Nowadays, the global incidence of DM is rapidly rising, more and more researchers have paid more attention to its associated complications. Statistics have shown that more than 15% of patients suffered from DM develop chronic non-healing wounds 4. The common wound healing is considered as a natural physiological process, including hemostasis, inflammation, proliferation and remodeling 23. However, the healing process in DW is usually disrupted by prolonged accumulation of inflammatory cells. Under pathological conditions, diabetic hyperglycemia triggers inflammatory cascades and lead to dysregulated angiogenesis, finally contributing to chronic non-healing wound.

Accumulating evidence suggested that naturally occurring polyphenol had remarkable health-prompting functions in a wide range of chronic diseases 24. QCT, a naturally polyphenolic flavonoid found abundantly in various vegetables and fruits, exhibits a range of biological activities including anti-inflammatory, anti-apoptotic, anti-oxidative, anti-infective, and anti-tumor effects. Additionally, it promotes vasodilation and has therapeutic potential for the treatment of metabolic and cardiovascular diseases 25. In recent years, the beneficial therapeutic roles of QCT in DW have been extensively discussed. A study by Latif reported that using a newly synthesized agent (QCT and its esterified complex with 4FPBA-Q) significantly accelerated wound healing in infected diabetic rats 26. A study by Aboud *et al.* found that QCT combined with laser therapy attenuated the expression of pro-inflammatory cytokines and accelerated the wound healing process 27. Besides, Asemi suggested that QCT was an effective polyphenol against DW and reviewed the effects of QCT for DW treatment. He suggested that the therapeutic effects of QCT for DW treatment might be related to its anti-inflammation and anti-oxidant properties 28. All these findings suggested that QCT was an effective and promising polyphenol against DW. In this study, we used network pharmacological to predict and screen the possible therapeutic targets and associated signaling pathways of QCT for treating DW. Additionally, experimental methodologies were utilized to uncover the potential mechanisms of action of QCT.

Through constructing a PPI network, we identified seven pivotal hub genes essential for treating DW with QCT. IL6, a cytokine involved in inflammation and immune responses, is crucial for initiating inflammation necessary for wound healing. However, excessive IL6 activity can lead to chronic inflammation, impairing the healing process 29, 30. MMP9, an enzyme that degrades extracellular matrix components, is involved in tissue remodeling and repair. In DWs, abnormally high MMP9 levels result in excessive matrix degradation, preventing new tissue formation and prolonging inflammation 31, 32. AKT1, a serine/threonine kinase, promotes cell proliferation, migration, and survival, all critical for wound healing. In diabetes, impaired AKT1 signaling reduces cellular responses necessary for effective wound healing, such as keratinocyte migration and angiogenesis 33. TNF, a cytokine regulating immune cells and systemic inflammation, is essential for initiating inflammation and mobilizing immune cells to the wound site. However, excessively high TNF levels in DWs contribute to chronic inflammation, tissue damage, and delayed healing 34, 35. EGFR, a receptor tyrosine kinase, activates pathways promoting cell growth, survival, and proliferation. EGFR signaling is crucial for keratinocyte migration, proliferation, and differentiation, necessary for wound re-epithelialization. In diabetes, impaired EGFR signaling leads to delayed wound closure and defective tissue repair 36. SRC, a non-receptor tyrosine kinase, regulates various cellular processes, including proliferation, differentiation, and survival. SRC influences cell migration and adhesion through responses to growth factors and integrins. Dysregulation of SRC activity in DWs impairs these processes, resulting in delayed healing and reduced tissue regeneration 37. Finally, c-Jun, a component of the transcription factor AP-1, regulates gene expression in response to stress and cytokines. c-Jun is involved in cell proliferation, differentiation, and apoptosis, and in DWs, dysregulated c-Jun activity can impair cell proliferation and delay wound closure 38. These hub genes play significant roles in the pathological processes affecting DW healing and are potential targets for therapeutic intervention.

Additionally, to elucidate the interactions between the target proteins and the bioactive compound, semiflexible molecular docking was conducted. The docking simulations revealed that QCT exhibited significant binding affinity towards the seven hub genes, with a particularly strong affinity for MMP9. The primary mode of interaction between QCT and the target proteins was identified as van der Waals forces.

In addition, the majority of hub genes were annotated to signaling pathways identified through GO and KEGG analyses, which are intricately associated with the wound healing process. GO enrichment analysis indicated that QCT is involved in key biological processes such as the positive regulation of cell motility and locomotion. KEGG pathway enrichment analysis demonstrated that QCT exerts therapeutic effects on DW by modulating several pathways, including the AGE-RAGE signaling pathway in diabetic complications, the IL-17 signaling pathway, the PI3K-Akt signaling pathway, and the TNF signaling pathway. The PI3K-AKT signaling pathway is integral to the wound healing process, particularly in regulating inflammation, cell proliferation, and tissue regeneration 39, 40. Activation of PI3Ks by growth factors and cytokines leads to the production of PIP3, which recruits and activates AKT through phosphorylation 41. AKT, in turn, influences numerous downstream targets, including the mammalian target of rapamycin (mTOR). This pathway modulates the inflammatory response by regulating the production of pro-inflammatory cytokines and growth factors, essential for the initial phase of wound healing 42. Additionally, AKT promotes the proliferation and migration of keratinocytes and fibroblasts, crucial for re-epithelialization and tissue formation, while mTORC1 enhances protein synthesis and cell growth, aiding in tissue repair and regeneration 43. The pathway also supports angiogenesis by upregulating vascular endothelial growth factor (VEGF), ensuring an adequate blood supply to the healing tissue. Furthermore, mTOR influences the production and remodeling of the extracellular matrix (ECM), providing structural support to new tissue 44. Activation of this pathway is essential for promoting angiogenesis. In human umbilical vein endothelial cells (HUVECs) exposed to high glucose conditions, there is a notable decrease in the expression of p-PI3K and p-AKT. Importantly, the angiogenic capacity and functionality of HUVECs can be significantly enhanced through the upregulation of the PI3K/AKT/eNOS pathway 45, 46. These findings suggest that targeting the PI3K-AKT pathway represents a promising therapeutic strategy for treating DWs.

In this study, QCT was chosen as the intervention for DW model rats to investigate its mechanism of action and validate the outcomes derived from network pharmacology analysis. Our data show that treatment with QCT significantly improved the wound healing rate compared with DW model group. In addition, WB results demonstrated that QCT treatment significantly reduced the protein levels of the inflammatory cytokines IL-6, TNF-α, and MMP9 at early stage. This reduction validates the therapeutic effect of QCT on DWs, as previously mentioned, where elevated levels of MMP9 and IL-6 were observed in DW tissues. Compared to the control group, the DW group exhibited a significant downregulation in the protein levels of p-AKT, p-PI3K, and mTOR. Conversely, these protein levels were significantly elevated in the QCT treatment group. Moreover, histological analysis indicated that after QCT treating, significantly increased collagen fiber deposition and the presence of blood vessels and hair follicles were observed, accompanied with decreased in the production of pro-inflammatory cytokines.

Our study suggests that QCT can treat DWs through interactions with multiple target proteins. These molecules play crucial roles in the complex process of wound healing, and their dysregulation in diabetes contributes to the chronicity and impaired healing characteristic of DWs. The therapeutic process of QCT on DW primarily involves activating the PI3K-AKT signaling pathway and reducing the inflammatory response.

## Conclusion

In this study, we used network pharmacology methods combined with molecular docking to identify therapeutic targets and pathways of QCT for treating DW. In view of our results, we identified 7 potential therapeutic targets (including MMP9, TNF-α, IL-6, EGFR, AKT1, SRC and JUN) of QCT in the treatment of DW. The *in silico* studies showed that QCT had better affinities with these targets. Furthermore, our results proved that QCT accelerated DW healing by reducing the activation of pro-inflammatory cytokines and regulating the PI3K-AKT-mTOR signaling pathway. Our study might provide theoretical basis for further investigation of QCT in DW treatment.

## Figures and Tables

**Table 1:** Putative genes of QCT for DW repairing.

No.	Gene	Uniprot ID	No.	Gene	Uniprot ID	No.	Gene	Uniprot ID
1	ABCB1	P08183	31	DPP4	P27487	61	MYLK	Q15746
2	ABCC1	P33527	32	DRD4	P21917	62	NCOA1	Q15788
3	ABCG2	Q9UNQ0	33	EGFR	P00533	63	NOS3	P29474
4	ACHE	P22303	34	ESR1	P03372	64	NOX4	Q9NPH5
5	ACTB	P60709	35	ESR2	Q92731	65	NQO1	P15559
6	ADORA1	P30542	36	ESRRA	P11474	66	NR1I2	O75469
7	ADORA2A	P29274	37	F2	P00734	67	PARP1	P09874
8	ADRB2	P07550	38	GPR35	Q9HC97	68	PIK3CG	P48736
9	AHR	P35869	39	GSK3B	P49841	69	PIK3R1	P27986
10	AKR1B1	P15121	40	HSP90AA1	P07900	70	PLAT	P00750
11	AKR1B10	O60218	41	IFNG	P01579	71	PLAU	P00749
12	AKT1	P31749	42	IGF1R	P08069	72	PON1	P27169
13	ALOX12	P18054	43	IL6	P05231	73	PPARG	P37231
14	ALOX15	P16050	44	INSR	P06213	74	PRKACA	P17612
15	ALOX5	P09917	45	JAK1	P23458	75	PRKCA	P17252
16	APEX1	P27695	46	JUN	P05412	76	PRKCB	P05771
17	APP	P05067	47	KCNH2	Q12809	77	PTGS1	P23219
18	AR	P10275	48	KDR	P35968	78	PTGS2	P35354
19	ARG1	P05089	49	MAPK1	P28482	79	PTK2	Q05397
20	AVPR2	P30518	50	MAPT	P10636	80	PTK2B	Q14289
21	AXL	P30530	51	MET	P08581	81	SELE	P16581
22	CCL2	P19875	52	MGAM	O43451	82	SHBG	P04278
23	CD38	P28907	53	MMP1	P03956	83	SRC	P12931
24	CDK2	P24941	54	MMP12	P39900	84	TERT	O14746
25	CDK5	Q00535	55	MMP13	P45452	85	TF	P02787
26	COMT	P21964	56	MMP2	P08253	86	THBD	P07204
27	CTSD	P07339	57	MMP3	P08254	87	TNF	P01375
28	CXCR1	P25024	58	MMP9	P14780	88	TTR	P02766
29	CYP19A1	P11511	59	MPO	P05164	89	VCAM1	P19320
30	DNMT1	P26358	60	MTTP	P55157	90	XDH	P47989

**Table 2:** Selection for hub genes.

Rank	MNC	MCC	Degree
1	ACTB	ACTB	AKT1
2	AKT1	AKT1	CCL2
3	EGFR	EGFR	EGFR
4	ESR1	IL6	ESR1
5	HSP90AA1	JUN	HSP90AA1
6	IL6	MMP9	IL6
7	JUN	PPARG	JUN
8	MMP9	PTGS2	MMP9
9	SRC	SRC	SRC
10	TNF	TNF	TNF

**Table 3:** Enriched biological processes relating to core targets.

Term	Biological process	Contained core genes	P value
GOBP:1901699	cellular response to nitrogen compound	EGFR, SRC, AKT1, TNF	1.00E-29
GOBP:0071417	cellular response to organonitrogen compound	EGFR, SRC, AKT1, TNF	1.00E-27
GOBP:0010035	response to inorganic substance	EGFR, SRC, AKT1, IL6, JUN, MMP9	1.00E-25
GOBP:0045937	positive regulation of phosphate metabolic process	EGFR, SRC, AKT1, IL6, MMP9, TNF	1.00E-23
GOBP:0010562	positive regulation of phosphorus metabolic process	EGFR, SRC, AKT1, IL6, MMP9, TNF	1.00E-23
GOBP:1904645	response to amyloid-beta	SRC, AKT1, TP53, EGFR, MMP9, TNF	1.00E-23
GOBP:1901652	response to peptide	AKT1, MMP9, SRC, TNF	1.00E-23
GOBP:2000147	positive regulation of cell motility	AKT1, EGFR, IL6, MMP9, SRC, TNF	1.00E-22
GOBP:0040017	positive regulation of locomotion	AKT1, EGFR, IL6, MMP9, SRC, TNF	1.00E-22
GOBP:0009725	response to hormone	AKT1, EGFR, IL6, SRC, TNF	1.00E-22
GOCC:0045121	membrane raft	EGFR, SRC, TNF	1E-16
GOCC:0098857	membrane microdomain	EGFR, SRC, TNF	1E-16
GOCC:0048471	perinuclear region of cytoplasm	EGFR, SRC	1E-10
GOCC:0030425	dendrite	SRC	3.98E-10
GOCC:0097447	dendritic tree	SRC	3.98E-10
GOCC:0043235	receptor complex	EGFR, IL6	7.94E-10
GOCC:0044853	plasma membrane raft	SRC	1.58E-09
GOCC:0005901	caveola	SRC	5.01E-09
GOCC:0031252	cell leading edge	AKT1, EGFR, SRC	5.01E-09
GOCC:0030424	axon	SRC	7.94E-09
GOMF:0004672	protein kinase activity	AKT1, EGFR, SRC	1E-17
GOMF:0016773	phosphotransferase activity	AKT1, EGFR, SRC	1E-16
GOMF:0016301	kinase activity	AKT1, SRC	1E-16
GOMF:0004713	protein tyrosine kinase activity	SRC	1E-11
GOMF:0017171	serine hydrolase activity	MMP9	1E-11
GOMF:0004252	serine-type endopeptidase activity	MMP9	1.26E-10
GOMF:0004879	nuclear receptor activity	-	2E-10
GOMF:0098531	ligand-activated transcription factor activity	-	2E-10
GOMF:0008236	serine-type peptidase activity	MMP9	3.16E-10
GOMF:0061629	RNA polymerase II-specific DNA-binding transcription factor binding	JUN, SRC	5.01E-10

**Table 4:** The top 20 enriched KEGG pathways.

Term	Pathway	Contained genes	P value
hsa05200	Pathways in cancer	AKT1, IL6, JUN, MMP9	1.00E-28
hsa05418	Fluid shear stress and atherosclerosis	AKT1, SRC, JUN, TNF, MMP9	1.00E-26
hsa05417	Lipid and atherosclerosis	AKT1, SRC, TNF, IL6, JUN	1.00E-22
hsa04933	AGE-RAGE signaling pathway in diabetic complications	AKT1, IL6, JUN, TNF	1.00E-21
hsa05205	Proteoglycans in cancer	AKT1, EGFR, SRC, TNF, MMP9	1.00E-21
hsa01521	EGFR tyrosine kinase inhibitor resistance	AKT1, SRC, EGFR, IL6	1.00E-21
hsa04915	Estrogen signaling pathway	AKT1, EGFR, JUN, SRC, MMP9	1.00E-19
hsa04657	IL-17 signaling pathway	TNF, MMP9, IL6, JUN	1.00E-18
hsa05215	Prostate cancer	AKT1, MMP9, EGFR	1.00E-18
hsa01522	Endocrine resistance	AKT1, EGFR, MMP9, SRC, JUN	1.00E-18
hsa05163	Human cytomegalovirus infection	AKT1, EGFR, SRC, TNF, IL6	1.00E-17
hsa04510	Focal adhesion	AKT1, EGFR, SRC, JUN	1.00E-16
hsa05207	Chemical carcinogenesis - receptor activation	AKT1, EGFR, JUN, SRC	1.00E-16
hsa04926	Relaxin signaling pathway	AKT1, EGFR, JUN, SRC, MMP9	1.00E-16
hsa04151	PI3K-Akt signaling pathway	AKT1, IL6, EGFR	1.00E-15
hsa04066	HIF-1 signaling pathway	AKT1, EGFR, IL6	1.00E-15
hsa04668	TNF signaling pathway	AKT1, IL6, JUN, MMP9, TNF	1.00E-15
hsa04921	Oxytocin signaling pathway	SRC, EGFR, JUN	1.00E-15
hsa04370	VEGF signaling pathway	AKT1, SRC	1.00E-15
hsa05161	Hepatitis B	AKT1, IL6, JUN, MMP9, SRC	1.00E-15

**Table 5:** Molecular docking score.

No	Target (PDB)	Binding sites with amino acid	Binding energy(kcal/mol)
1	MMP9 (4hma)	PRO254, HIS257, PRO255, LEU243, TYR245, THR251, GLU241	-7.35
2	TNF (1a8m)	GLY24, ALA134, TRP28, ASN46, LEU26, GLN25	-5.48
3	IL6 (1alu)	GLU93, THR138, THR137, THR143, ASP140, PRO139, ASN63	-4.26
4	EGFR (7u99)	LEU844, ALA743, VAL726, MET793, LEU718, GLY796, CYS797, ASP800	-4.26
5	AKT (2uzs)	LYS17, THR87, ARG86, ARG15, LYS20	-4.16
6	SRC (1y57)	GLN324, LEU325, TRP260, ARG318, MET314, LEU317, LYS315	-3.85
7	JUN (5fv8)	GLN30, MET26, PRO37, GLN33, GLU19, SER22	-3.75
